# Beating the Odds: A Successful Pregnancy in a Patient With ALK-Rearranged Lung Cancer on Alectinib

**DOI:** 10.1155/carm/9032427

**Published:** 2025-05-10

**Authors:** Nicholas Yeo, Fiona Johnston, Louise Fay, Paul Lilburn, Debra Kennedy, Helen Barrett, Antonia Shand, Giselle Kidson-Gerber, Monica Tang, Daniel Challis, Benjamin Y. Kong

**Affiliations:** ^1^Department of Medical Oncology, Prince of Wales Hospital, Nelune Comprehensive Cancer Centre, Randwick, Australia; ^2^School of Clinical Medicine, University of New South Wales, Sydney, Australia; ^3^Department of Obstetrics & Gynaecology, The Royal Hospital for Women, Randwick, Australia; ^4^Department of Respiratory Medicine, Prince of Wales Hospital and Community Health Services, Randwick, Australia; ^5^Faculty of Medicine and Health, The University of Sydney, Sydney, Australia

## Abstract

The management of oncogene-driven non–small cell lung cancer (NSCLC) during pregnancy presents unique challenges due to limited safety data on targeted therapies. Anaplastic lymphoma kinase (ALK)-rearranged NSCLC is a rare but increasingly recognized entity in young women, including during pregnancy. Here, we report the case of a 37-year-old woman diagnosed with metastatic ALK-rearranged NSCLC during her first pregnancy, who was commenced on alectinib. Following this, she had a successful second pregnancy whilst being treated with alectinib (withheld during weeks 6–10 of gestation to avoid the critical period of organogenesis). Both pregnancies resulted in healthy infants with no complications or evidence of developmental delays. This case highlights the importance of a multidisciplinary approach involving oncology, maternal-fetal medicine, clinical genetics, obstetrics medicine, and obstetrics to balance maternal cancer control and fetal health. While preclinical studies of alectinib suggest teratogenic risks, this and other reported cases demonstrate its potential for safe use during pregnancy with careful planning. Pathological examination of the placenta in our case revealed no malignant cells, and maternal disease remained controlled. As targeted therapies extend survival in advanced NSCLC, more patients may contemplate pregnancy, emphasizing the need for robust evidence to guide treatment decisions. This case contributes to the growing body of evidence supporting the feasibility of managing pregnancy in patients with ALK-rearranged NSCLC using targeted therapies like alectinib, while underscoring the importance of long-term follow-up for the exposed offspring.

## 1. Introduction

The incidence of cancer during pregnancy is estimated at approximately 1 in 1000 pregnancies [1], with an increasing trend in the rate of pregnancy-associated cancers [2–4]. This is relatively rare but has become more common in developed countries due to the trend of delayed childbearing [1]. Whilst a diagnosis of non‐small cell lung cancer (NSCLC) in pregnancy is rare, anaplastic lymphoma kinase (ALK)-rearranged NSCLCs accounts for 38% of NSCLC in women of childbearing age [5]. Moreover, ALK-rearranged NSCLCs account for 12.5% of NSCLC diagnosed in pregnancy [5]. Due to advances in the treatment of oncogene-addicted NSCLC, patients with historically guarded prognosis are now able to live much longer [6, 7], raising the possibility of childbearing [8, 9]. The management of cancer in pregnant women is complex, however, due to the need to balance effective anticancer therapies whilst minimizing risks to the fetus [1, 9].

Chemotherapeutic agents are generally contraindicated during the first trimester, to avoid the critical period of organogenesis [10]. Targeted treatments, including tyrosine kinase inhibitors (TKIs) such as alectinib on the other hand have limited data to guide administration during pregnancy. According to the Food and Drug Administration (FDA) pharmacology report, alectinib caused severe embryofetal toxicities in rats at maternally toxic oral exposures that were 2.7 times the human exposure and complete litter loss at 4.5 times the human exposure [11]. To date, there are only a handful of case reports describing patients treated with TKIs during pregnancy for ALK-rearranged NSCLC [12–18].

Here, we describe the case of a patient with an ALK-rearranged metastatic NSCLC who had a successful planned pregnancy whilst being treated with alectinib.

## 2. Case Report

**Figure 1:**
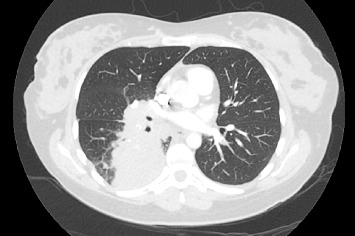
CTPA at diagnosis showing right lower lobe mass with extensive hilar and mediastinal lymphadenopathy.

A 37-year-old woman at 37 weeks gestation of her first pregnancy presented to hospital with dyspnea, cough, and right-sided pleuritic chest pain. A computed tomography pulmonary angiogram (CTPA) showed bilateral pulmonary emboli (PE) and a right lower lobe mass with extensive hilar and mediastinal lymphadenopathy (Figure 1). She was commenced on enoxaparin for her PE. This was transitioned to a heparin infusion in preparation for the birth of her baby. She had a vacuum assisted vaginal delivery with no significant complications.

**Figure 2:**
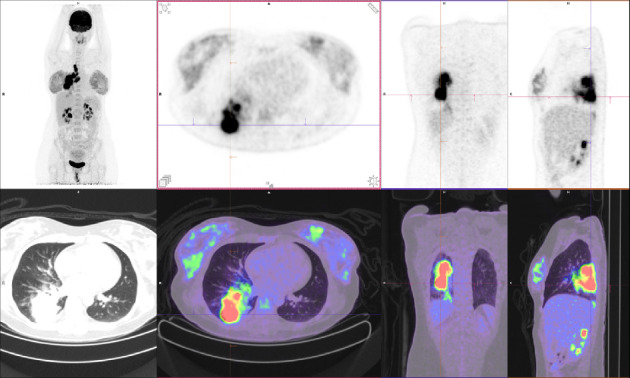
FDG PET-CT at diagnosis.

Subsequently, an endobronchial ultrasound-guided transbronchial needle aspiration (EBUS-TBNA) of the right hilar 10R and station 7 lymph nodes were performed. Histopathology revealed malignant cells positive for TTF1, napsin A, and CK7 and negative for CK20, consistent with an adenocarcinoma of the lung. Immunohistochemistry showed diffuse 3+ positive staining for ALK, and fluorescent in situ hybridization assay (FISH) confirmed an ALK rearrangement. Programmed death-ligand 1 (PD-L1) tumor proportion score (TPS) was 20%. An [^18^F]-fluorodeoxyglucose positron emission tomography-CT (FDG PET-CT) was performed, showing an FDG-avid primary lesion in the right lower lobe of the lung with extensive ipsilateral para-hilar and mediastinal nodal metastatic disease (Figure 2). A magnetic resonance imaging (MRI) brain showed no intracranial metastases. Consensus from the lung multidisciplinary meeting (MDM) was that her disease was neither resectable nor suitable for curative intent chemoradiation. She was therefore commenced on alectinib 600 mg twice daily, a 2^nd^-generation ALK TKI.

**Figure 3:**
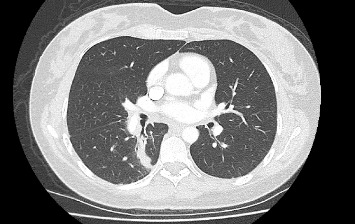
CT chest 3 months after treatment with alectinib, showing a significant reduction in the size of the right lower lobe lung lesion.

Progress imaging 3 months later showed a significant reduction in the size of her right lower lobe mass and associated hilar and mediastinal lymphadenopathy (Figure 3).

A year later, the patient and her partner considered a second pregnancy. The lack of available literature on treatment with alectinib during pregnancy was discussed. Teratogenicity from alectinib in murine studies in preclinical literature was conveyed. The changes in drug pharmacodynamics in pregnancy, effect on fertility, in-utero exposure to drug, and theoretical risk of malignant cell transfer across the placenta were also highlighted. Furthermore, the prognosis from her incurable lung cancer in the context of planning for a second pregnancy was also discussed. The patient weighed these considerations and ultimately decided to proceed with a second pregnancy, accepting the various risks discussed. Clinical genetics, maternal-fetal medicine, obstetrics medicine, and obstetrics were consulted—a recommendation was made to cease alectinib between weeks 6–10 of pregnancy to avoid the high-risk period of organogenesis. She conceived a month later. She was commenced on thromboprophylaxis with enoxaparin 40 mg daily given her previous PE. This was continued throughout pregnancy and continued for 6 weeks postpartum. She underwent a vaginal delivery at 39.1 weeks gestation. The baby weighed 3.39 kg (50–75^th^ centile) and had Apgar scores of 9 at 5 min. There were no complications. Pathological examination of the placenta showed no evidence of malignant cells or chorioamnionitis. As data from maternal-fetal medicine indicated that there is potentially a small amount of alectinib expressed in breast milk, the patient elected for bottle feeding.

She continues on alectinib with progress imaging showing ongoing disease control. Her child continues to show normal development at 8 months.

## 3. Discussion

Our case illustrates the complexities in the care of a pregnant patient with an oncogene-addicted NSCLC, where there is limited safety data on the use of targeted agents. Data in this setting are limited to case reports (Table 1). In these reports, treatment with alectinib did not compromise pregnancy, fetal, or developmental outcomes [12–17]. Pathological examination of the placenta did not show any evidence of malignancy [13, 15].

We found two studies which had measured alectinib concentrations. Scarfone and colleagues described a patient who continued alectinib throughout pregnancy, who gave birth to a healthy infant at 35 weeks of gestation [16]. At birth, maternal plasma levels of alectinib were noted to be 14 times higher than that in the infant. The placental concentration of alectinib was 562 ng/g. In another patient treated with alectinib throughout her pregnancy, alectinib concentration in maternal blood was 155 ng/mL at birth, 22.1 ng/mL in the umbilical cord venous blood, 20.1 ng/mL in amniotic fluid, and 11.8 ng/mL in the colostrum [14]. In both cases, the higher maternal blood concentration of alectinib and high concentration of alectinib in the placenta suggest a considerable separation and filtering of alectinib by the placental barrier.

Pregnancy in patients with active or previous cancers requires careful balancing of competing priorities, that is, to minimize the effects of the cancer and cancer treatments on the health of the pregnant mother and fetus, while also mitigating any potential impact of pregnancy on the risk of cancer progression or recurrence. The bulk of existing research on pregnancy in cancer patients have focused on long-term survivors of childhood or young adult cancers, who are typically considered cured [19]. These demonstrate no evidence of increased congenital or chromosomal abnormalities in childhood cancer survivors [20–22] but mixed results regarding increased risk of pregnancy-related complications and birth outcomes depending on the type of prior cancer treatment [23–27]. However, there are few studies of pregnancy in patients with cancer on active treatment, and our study adds to the evidence that TKI treatment can be safely managed to facilitate conception, gestation, and delivery.

As cancer treatments improve and the prospect of long-term disease control and even cure becomes feasible in traditionally “incurable” cancers, such as metastatic melanoma and oncogene-driven NSCLCs, pregnancy will become more frequently contemplated in patients with advanced cancers. A key consideration is whether pregnancy, which may necessitate interruption of cancer treatment, will increase risks of cancer progression or recurrence. For patients with hormone receptor-positive early breast cancer, the POSITIVE trial demonstrated that temporary interruption of endocrine therapy to attempt pregnancy did not confer a greater risk of breast cancer recurrence [28]. Unfortunately, similar large prospective studies are unlikely to be conducted in patients with oncogene-driven NSCLCs due to much smaller patient populations. Moreover, the complexities of family planning in a patient with an incurable cancer albeit median overall survival of > 5 years are highlighted in our case [7]. Therefore, case reports such as this will be pivotal in building the body of evidence to guide clinical decision-making and management around pregnancy in this cohort.

## 4. Conclusion

The management of pregnant patients with oncogene-driven NSCLC presents significant challenges due to the lack of robust safety data on targeted therapies and the complex interplay between maternal cancer control and fetal health. This case underscores the importance of a multidisciplinary approach that integrates oncology, maternal-fetal medicine, clinical genetics, and obstetrics to navigate these complexities. Although targeted therapies such as alectinib have shown promising efficacy in controlling disease, their use during pregnancy requires cautious planning, particularly around critical periods like organogenesis. In this case, we observed no adverse fetal or maternal outcomes, reinforcing the potential for successful pregnancy management in carefully selected patients. However, long-term follow-up is essential to fully understand the developmental outcomes for children exposed to targeted therapies in utero. As oncogene-driven cancers become increasingly manageable and patients live longer, more research is needed to guide treatment and pregnancy planning in this evolving therapeutic landscape.

## Figures and Tables

**Table 1:** Case reports of patients with ALK-rearranged NSCLC who have received ALK TKIs during pregnancy.

Author	Treatment	Alectinib exposure period	Pregnancy: planned versus unplanned	Pregnancy outcomes	Fetal outcomes
Gonzalez-Mosquera et al. [12]	Alectinib 600 mg BD	18–39 weeks	Unplanned	Elective induction of labor at 39 weeks	Normal development at 2 months

Wu et al. [13]	Alectinib 600 mg BD	Continued throughout pregnancy	Unplanned	Elective caesarean section at 38 weeks	Normal development at 26 months

Kato et al. [14]	Alectinib 600 mg BD	Continued throughout pregnancy	Unplanned	Spontaneous vaginal delivery at 41 weeks	Normal development at 13 months

Weidenbaum et al. [15]	Alectinib 300 mg BD	Continued throughout pregnancy	Two separate pregnancies: unplanned (pregnancy 1) and planned (pregnancy 2)	Pregnancy 1: elective induction of labor at 39 weeks	Normal development at 33 months and 10 months respectively
				Pregnancy 2: spontaneous vaginal delivery at 39 weeks	

Scarfone et al. [16]	Alectinib 600 mg BD	Continued throughout pregnancy	Unplanned	Elective caesarean section at 35 weeks	Normal development at 19 months

De Smedt et al. [17]	Alectinib 600 mg BD	Continued throughout pregnancy	Unplanned	Elective induction of labor at 36 weeks	NA

## Data Availability

Data sharing is not applicable to this article as no new data were created or analyzed in this study.
